# Enhancement of acetoin production in *Candida glabrata* by *in silico*-aided metabolic engineering

**DOI:** 10.1186/1475-2859-13-55

**Published:** 2014-04-13

**Authors:** Shubo Li, Xiang Gao, Nan Xu, Liming Liu, Jian Chen

**Affiliations:** 1 State Key Laboratory of Food Science and Technology, Jiangnan University, Wuxi, Jiangsu 214122, China; 2 The Key Laboratory of Industrial Biotechnology, Ministry of Education, Jiangnan University, Wuxi 214122, China; 3 Laboratory of Food Microbial-Manufacturing Engineering, Jiangnan University, Wuxi 214122, China

**Keywords:** Acetoin, *Candida glabrata*, Cofactor engineering, Heterologous pathway, *In silico*, Metabolic engineering

## Abstract

**Background:**

Acetoin is a promising chemical compound that can potentially serve as a high value-added platform for a broad range of applications. Many industrial biotechnological processes are moving towards the use of yeast as a platform. The multi-auxotrophic yeast, *Candida glabrata*, can accumulate a large amount of pyruvate, but produces only trace amounts of acetoin. Here, we attempted to engineer *C. glabrata* to redirect the carbon flux of pyruvate to increase acetoin production.

**Results:**

Based on an *in silico* strategy, a synthetic, composite metabolic pathway involving two distinct enzymes, acetolactate synthase (ALS) and acetolactate decarboxylase (ALDC), was constructed, leading to the accumulation of acetoin in *C. glabrata*. Further genetic modifications were introduced to increase the carbon flux of the heterologous pathway, increasing the production of acetoin to 2.08 g/L. Additionally, nicotinic acid was employed to regulate the intracellular NADH level, and a higher production of acetoin (3.67 g/L) was obtained at the expense of 2,3-butanediol production under conditions of a lower NADH/NAD^+^ ratio.

**Conclusion:**

With the aid of *in silico* metabolic engineering and cofactor engineering, *C. glabrata* was designed and constructed to improve acetoin production.

## Background

Acetoin (3-hydroxy-2-butanone) is known to be a major volatile compound, naturally present in wine, cocoa, butter, honey, coffee, strawberries, etc. Additionally, as a member of the C4-dicarboxylic acid family, acetoin was also defined as one of the potential top 30 sugar-derived chemical building blocks by the U.S. Department of Energy [1], and has drawn much interest because it could serve as a high value-added platform for the food, flavor, cosmetics, pharmaceutical, and chemical industries [2,3]. At present, three methods are used to produce acetoin: microbial fermentation, enzymatic conversion, and chemical synthesis [4]. Of those, microbial fermentation is the more cost-effective approach owing to the low cost of raw materials, mild process conditions, and high purity of product [5,6]. However, acetoin is only a minor by-product in mixed acid fermentation in a number of microorganisms, such as *Lactococcus lactis*[5], *Bacillus subtilis*[6], *Klebsiella pneumonia*[7], and *Bacillus amyloliquefaciens*[8]. In addition, because of the issues of safety, microbial robustness, and phage contamination, industrial-scale production of acetoin has not yet been realized using these strains. Therefore, the biological platform for microbial acetoin production needs to be further refined and improved.

Recently, many industrial biotechnological processes are moving towards the use of yeast as a platform because it can withstand lower temperatures and is associated with easier separation of products, lack of phage contamination, ease of scale-up, lower pH tolerance, and greater robustness [9]. However, only small quantities of acetoin (< 500 mg/L) can be accumulated in wild-type yeast [10]. Furthermore, some factors, including the lack of extensive genetic and metabolic information, the selection of targeted genes, and the unpredictability of cellular physiological responses, have inhibited and raised some challenges for attempts to metabolically engineer improved cellular phenotypes of yeast in the post-genomic era [11,12]. To this end, the genome-scale metabolic models (GSMMs) of microorganisms, such as *E. coli*[13], *S. cerevisiae*[14], and *Candida glabrata*[15], have been reconstructed and used to identify genes that could be targeted for improving strains on a global level. With the aid of *in silico* simulation, systems metabolic engineering has opened a novel avenue for engineering microorganisms to produce value-added products [16,17].

**Figure 1:**
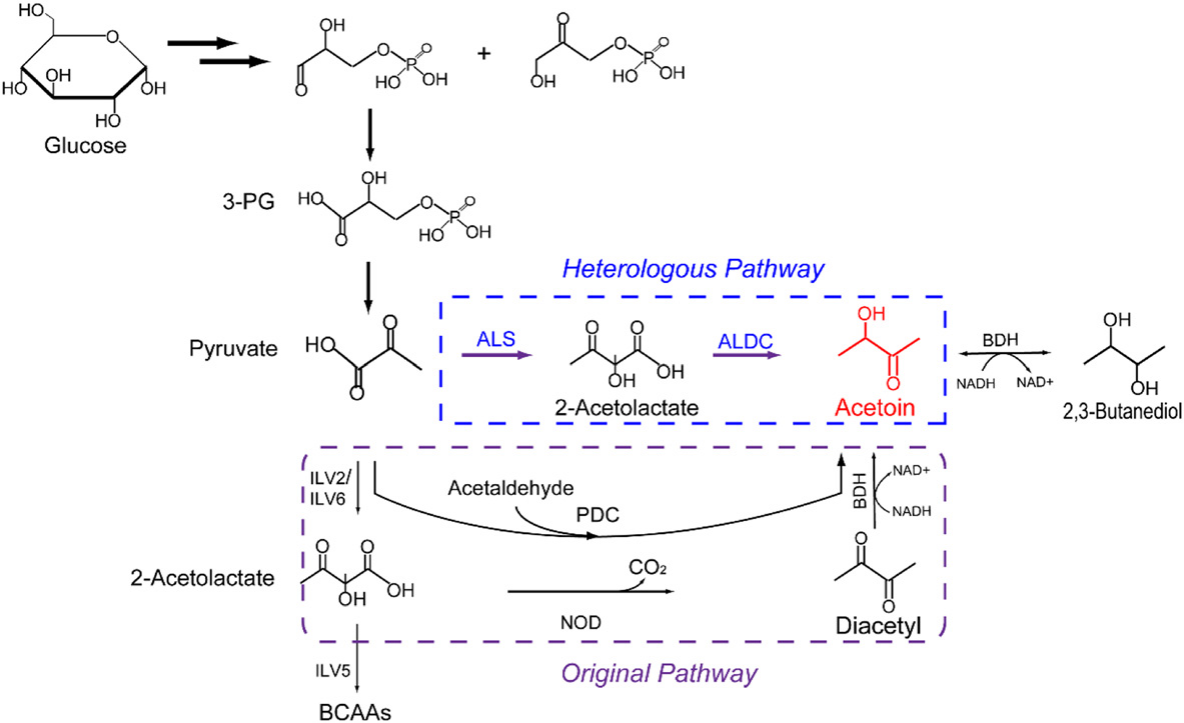
**Metabolic pathways of acetoin in *C. glabrata*: the original and heterologous metabolic pathways. **The enzymes that catalyze the pathways were: acetolactate synthase (ILV2 + ILV6, ALS), acetolactate decarboxylase (ALDC), pyruvate decarboxylase (PDC); butanediol dehydrogenase (BDH), acetohydroxy acid isomeroreductase (ILV5). The abbreviations are: BCAAs, branched chain amino acids; 3-PG, 3-phosphate-glyceraldehyde; NOD, non-enzymatic oxidative decarboxylation.

As a multi-auxotrophic yeast, *C. glabrata* can accumulate a large amount of pyruvate, providing an abundance of precursor for acetoin production [18]. With the availability of a fully sequenced genome and the reconstructed metabolic model *i* NX804, engineering *C. glabrata* can now be achieved more effectively and directly. However, owing to the inhibition of pyruvate decarboxylation and the intense spontaneous reaction of non-enzymatic oxidative decarboxylation (NOD), only 45 mg/L acetoin was accumulated in wild-type *C. glabrata* . Therefore, constructing a heterologous pathway in the cytosol may be the better option for acetoin production. As shown in Figure 1, the heterologous pathway converts pyruvate, the end metabolite of the glycolysis pathway, into acetoin via two enzymatic steps, which are catalyzed by α-acetolactate synthetase (ALS) [E.C.2.2.1.6] and α-acetolactate decarboxylase (ALDC) [E.C.4.1.1.5]. In this work, with the aid of *in silico* simulation, the heterologous pathway was designed and constructed for acetoin production in *C. glabrata* and, meanwhile, a series of genetic modifications were also applied to further increase acetoin production, demonstrating that *C. glabrata* is a promising candidate for production of acetoin on an industrial scale.

## Results

### Identification of the optimum pathway for acetoin production

**Figure 2:**
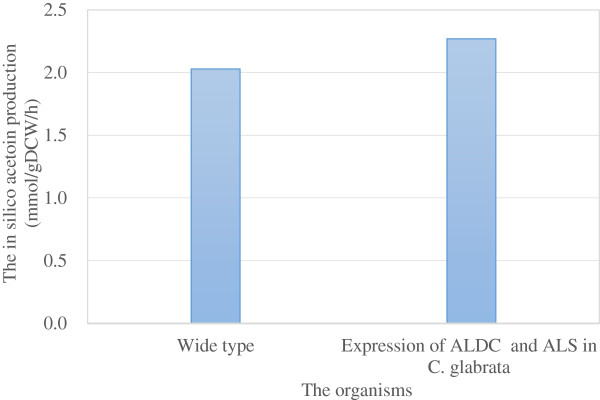
**Calculation of the heterologous pathway for acetoin production by *in silico *simulation.** The production of acetoin was simulated based on 10 mmol/gDCW/h of glucose uptake from two angles: setting cell growth as the objective function or maximizing acetoin production under 90% of the maximum μ.

By extensive mining of the literature and from the sequenced genome, two pathways for acetoin production, including pyruvate decarboxylation and non-enzymatic oxidative decarboxylation, were originally annotated in *C. glabrata*[10]. Considering the characteristics of *C. glabrata*, the production of acetoin was zero setting cell growth as the objective equation. To realize acetoin accumulation in *C. glabrata*, its metabolic capacity was then engineered by introducing a heterologous pathway in the cytosol, and evaluated by *in silico* simulation. According to the approach previously described [19-21], the reactions representing the enzymatic activities of α-acetolactate synthetase (ALS) and α-acetolactate decarboxylase (ALDC) were added into the model *i* NX804 prior to flux balance analysis, using acetoin as the objective equation by the FBA algorithm (Additional file 1a). As a result, the theoretical production rate of acetoin increased by 0.24 mmol/g DCW/h from 2.03 mmol/g DCW/h of acetoin, was accumulated when optimizing the biomass formation (Figure 2). Therefore, the *in silico* simulation showed that acetoin production could be enhanced by the additional reactions of ALS and ALDC, demonstrating that the heterologous pathway could be the better choice for acetoin production. Correspondingly, two genetic manipulation steps were carried out to implement the desired flux network in *C. glabrata*.

### Construction and optimization of the heterologous pathway

To construct the heterologous pathway, four different sources of ALS and ALDC were individually expressed in *C. glabrata* on a high copy plasmid using *TPI* promoter. In four ALS constructs, the highest values of ALS activity (0.81 U/mg protein from *B. subtilis*) and α-acetolactate concentration (0.70 g/L) were observed in strain MuA3, which were, respectively, 5.4-fold and 6.5-fold higher than the values observed for MuA0. However, the production of acetoin increased only slightly to 0.077 g/L, which was far below the production of α-acetolactate (Table 1). Additionally, diacetyl production also increased to 0.45 g/L from 0.02 g/L in strain MuA3. Thus, a suitable ALDC needed to be introduced for acetoin production. As shown in Table 1, because of the over-expression of ALDC, the highest ALDC specific activity was increased to 1.85 U/mg protein (from *B. amyloliquefaciens*) in strain MuA5. As a result, the production of acetoin increased to 0.14 g/L but no diacetyl was observed, indicating that α-acetolactate was completely decarboxylated, and became the rate-limiting step for acetoin production in ALDC constructs.

**Table 1:** **Effects of gene over-expression on the enzymes and metabolites**^**a**^

**Strains**	**Gene**	**Species**	**ALS**	**α-acetolactate**	**Diacetyl**	**ALDC**	**Acetoin**
			**(U/mg protein)**	**(g/L)**	**(g/L)**	**(U/mg protein)**	**(g/L)**
MuA0	nt	nt	0.15 ± 0.01	0.11 ± 0.01	0.02 ± 0.00	ND	0.041 ± 0.002
MuA1	*alsS*	*B. amyloliquefaciens*	0.75 ± 0.05	0.58 ± 0.02	0.37 ± 0.02	ND	0.054 ± 0.003
MuA2	*alsS*	*B. pumilus*	0.60 ± 0.05	0.47 ± 0.03	0.33 ± 0.03	ND	0.053 ± 0.001
MuA3	*alsS*	*B. subtilis*	0.81 ± 0.05	0.70 ± 0.05	0.45 ± 0.02	ND	0.077 ± 0.004
MuA4	*alsS*	*E. coli*	0.77 ± 0.1	0.62 ± 0.04	0.42 ± 0.04	ND	0.065 ± 0.002
MuA5	*alsD*	*B. amyloliquefaciens*	0.15 ± 0.01	0.11 ± 0.01	ND	1.85 ± 0.05	0.14 ± 0.04
MuA6	*alsD*	*B. pumilus*	0.14 ± 0.012	0.11 ± 0.01	ND	1.66 ± 0.05	0.12 ± 0.03
MuA7	*alsD*	*B. subtilis*	0.15 ± 0.02	0.12 ± 0.02	ND	1.71 ± 0.05	0.13 ± 0.06
MuA8	*alsD*	*E. coli*	0.13 ± 0.01	0.10 ± 0.02	ND	1.57 ± 0.1	0.12 ± 0.02

^a^The engineered strains were grown in fermentation medium, and the production of metabolites in the culture supernatant was determined by HPLC at 64 h during batch flask culture. Results are the average of three replicates with error bars indicating standard error from the mean. *ND*, Not detected; *nt*, Not.

**Figure 3:**
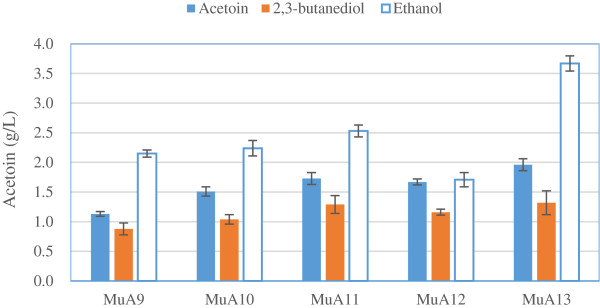
**Improvements in the production of acetoin, 2,3-butanediol and ethanol by using different promoters. **Results are the average of three replicates with error bars indicating standard error from the mean (MuA9 (P_TPI+TPI_), MuA10 (P_GPD+GPD_), MuA11 (P_TEF+TEF_), MuA12 (P_GPD+TEF_), MuA13 (P_TEF+GPD_)).

Accordingly, the best performing *ALS* and *ALDC* were co-expressed to construct the heterologous pathway in strain MuA9, in which the production of acetoin was increased to 1.14 g/L (Table 2). However, the accumulation of pyruvate was greater than the level of acetoin by 21-fold, giving 24 g/L. To address the bottleneck, a push-and-pull strategy that combines the amplification of an upstream pathway, with a similar increase in the flux of a downstream pathway, was adopted for strain improvement via the optimization of promoters. As shown in Figure 3, three promoters (P_*TPI*_, P_*GPD*_, P_*TEF*_) were selected for study, and it was found that suitable promoters had a positive effect on the production of acetoin. Compared to the control (MuA9), the highest specific activities of ALS and ALDC were obtained in strain MuA13 (harboring plasmid pY26-TEF- *ALS-* GPD- *ALDC*), in which they increased to 1.05 and 2.06 U/mg protein, respectively, due to the optimization of promoter combinations (Table 3). And correspondingly, the titer of acetoin also significantly increased, reaching 1.96 g/L, which was, respectively, 73.5%, 29.8%, 13.3%, and 17.4% higher than that of strains MuA9, MuA10, MuA11, and MuA12. Additionally, the highest yields of 2,3-butanediol and ethanol were obtained in strain MuA13, reaching 1.32 g/L and 3.72 g/L, which were, respectively, 50.0% and 73.0% higher than those of strain MuA9. Through enhancing the activities of ALS and ALDC, more carbon flux of pyruvate could be driven into the novel pathway for acetoin production.

**Table 2:** The comparison of fermentation characteristics of the engineered strains MuA0 and MuA9

**Parameters**^**a**^	**Strains**		**Fold change, B/A-1**
	**MuA0 (A)**	**MuA9 (B)**	
DCW (g/L)	10.84 ± 0.6	10.34 ± 0.5	-0.05
Pyruvate (g/L)	30.7 ± 1.2	24.4 ± 0.8	-0.20
Specific activity of ALS (U/mg protein)	0.16 ± 0.01	0.83 ± 0.04	4.19
Specific activity of ALDC (U/mg protein)	ND	1.86 ± 0.03	--
Acetolactate (g/L)	0.12 ± 0.01	0.74 ± 0.01	5
Acetoin (g/L)	0.042 ± 0.008	1.14 ± 0.05	26
Acetoin yield (g/g DCW)	0.003	0.11	35
2,3-Butanediol (g/L)	0.035 ± 0.004	0.88 ± 0.04	24
Diacetyl (g/L)	0.01 ± 0.00	ND	--
Ethanol (g/L)	0.87 ± 0.02	2.15 ± 0.2	1.47
Glycerol (g/L)	0.66 ± 0.04	1.15 ± 0.1	0.74

^a^Results are the average of three replicates with error bars indicating standard error from the mean. *ND*, Not detected.

**Table 3:** Effects of different genetic modifications on acetoin fermentation

Parameters*	**Strains**		
	**MuA13**	**MuA14**	**MuA15**
DCW (g/L)	10.27 ± 0.5	9.96 ± 0.4	10.13 ± 0.5
Pyruvate (g/L)	18.4 ± 0.7	16.8 ± 0.8	16.3 ± 1.1
**α**-acetolactate	0.72 ± 0.02	0.81 ± 0.01	0.74 ± 0.02
Specific activity of ALS (U/mg protein)	1.05 ± 0.03	1.07 ± 0.05	1.04 ± 0.04
Specific activity of ALDC (U/mg protein)	2.06 ± 0.06	2.03 ± 0.05	2.10 ± 0.1
Acetoin (g/L)	1.96 ± 0.3	2.13 ± 0.3	2.08 ± 0.2
Acetoin yield (g/g DCW)	0.19	0.21	0.21
2,3-Butanediol (g/L)	1.36 ± 0.03	1.45 ± 0.07	2.03 ± 0.03
Acetoin/2,3-Butanediol	1.44	1.47	1.02
Ethanol (g/L)	3.67 ± 0.11	2.96 ± 0.13	2.72 ± 0.2
Acetate (g/L)	0.64 ± 0.05	0.51 ± 0.05	0.83 ± 0.1
Glycerol (g/L)	1.01 ± 0.1	1.07 ± 0.12	1.12 ± 0.12
Intracellular NAD^+^ (mg/g DCW)	16.1 ± 1.2	14.6 ± 1.7	15.8 ± 2.1
Intracellular NADH/NAD^+^ ratio	0.62	0.61	0.64

^*^The production of metabolites in the culture supernatant was determined by HPLC at 64 h during flask culture. Results were the average of three replicates with error indicating standard error from the mean.

### Comparison of intracellular and extracellular acetoin

To evaluate the suitability of *C. glabrata* as a host for acetoin production, the ability to transport acetoin into the culture supernatant was investigated through determining the ratio of intracellular to extracellular acetoin production. In a defined number of cells, the amount of intracellular acetoin was compared to the accumulation of extracellular acetoin, which was produced and exported by those cells in shake-flask fermentation with strain MuA13. As a result, the intracellular and extracellular acetoin production reached 32 mg/L and 2.04 g/L, respectively, and a ratio of 1:65 ± 6 (intracellular: extracellular, data represent the average of biological duplicates) was obtained, indicating that *C. glabrata* could efficiently transport acetoin into the culture supernatant, and perform as an attractive host for acetoin production.

### Refinement of the completed pathway for acetoin production

In yeast, deleting the *ilv5* gene (encoding AHAIR) can block the subsequent step in the metabolism of α-acetolactate, leading to the leakage of α-acetolactate from the mitochondria and an increase in the availability of cytosolic α-acetolactate [22]. Therefore, the gene of *ilv5* (CAGL0B03047g) was deleted to generate strain MuA14. Compared to strain MuA13, the productions of acetoin and 2,3-butaediol were slightly increased to 2.13 g/L and 1.45 g/L, higher by 8.6% and 13.2%, respectively, but the cell growth decreased from 10.27 g/L to 9.96 g/L (Table 3). These results indicate that the deletion of *ilv5* had a slight effect on acetoin production at the expense of growth retardation *.* Furthermore, to decrease the formation of ethanol, the *ADH* gene (CAGL0J01441g) was disrupted on the basis of strain MuA13. As expected, ethanol production decreased to 2.72 g/L in strain MuA15, which was 34.9% less than that produced by MuA13. But interestingly, the levels of 2,3-butanediol and the NADH/NAD^+^ ratio increased to 2.03 g/L and 0.64, which were, respectively, 53.8% and 6.4% higher than in MuA13, but the production of acetoin (2.08 g/L) and the cell growth (10.13 g/L) were similar to those of MuA13 (Table 3). Thus, blockage of the ethanol pathway could enhance the carbon flux of the heterologous pathway, but was more favorable for forming 2,3-butanediol. Compared to strains MuA13 and MuA14, MuA15 has more potential and is more effective in redistributing the carbon flux of pyruvate, and was then selected to further improve acetoin production.

### Effect of nicotinic acid (NA) on acetoin production in a 5-L fermenter

**Figure 4:**
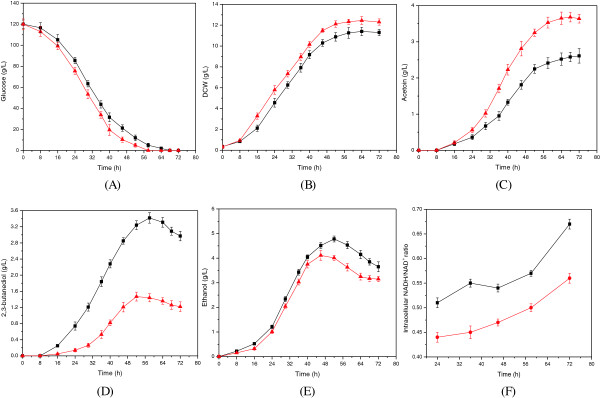
**Fermentation profiles for the control without the addition of NA (■) and with the addition of NA (▲) in a 5-L fermenter with MuA15. **The concentrations of glucose **(A)**, DCW **(B)**, acetoin **(C)**, 2,3-butanediol **(D)**, and ethanol **(E)**, and the intracellular NADH/NAD^+^ ratio** (F) **were measured and shown. The values were the mean derived from three independent biological experiments, and error bars indicated the standard deviations.

Based on the above results, it is suggested that the higher level of NADH/NAD^+^ was responsible for the increased 2,3-butanediol production in strain MuA15. To further increase acetoin production, 10 mg/L of NA (the precursor of NAD^+^) was added to regulate the level of NADH/NAD^+^ based on a previous study [23]. As shown in Figure 4F, the intracellular NADH/NAD^+^ ratio continuously increased during the acetoin fermentation, but a lower level of NADH/NAD^+^ was obtained due to the addition of NA, so that the level of intracellular NADH/NAD^+^ had decreased to 0.56 from 0.67 at the end of the acetoin fermentation. As a result, a series of changes were generated compared to the control (without addition of NA) (Figure 4), as follows. (1) Owing to the addition of NA, the strain showed a higher growth rate, in which the cell concentration and glucose consumption rate were increased by 16.9% and 17.6%, reaching 12.4 g/L DCW and 2.07 g/(L · h), respectively. (2) And correspondingly, the production and productivity of acetoin were increased to 3.67 g/L and 0.05 g/(L · h) by 40.6% and 38.9%, respectively, but the titer and productivity of 2,3-butanediol were significantly decreased to 1.22 g/L and 0.02 g/(L · h), which were, respectively, 118% and 115% lower than those of the control. Furthermore, the production of ethanol was also slightly decreased by 15% to 3.09 g/L. Thus, the addition of NA could effectively decrease the level of intracellular NADH/NAD^+^, and then significantly inhibit the degradation of acetoin and the biosynthesis of 2,3-butanediol, increasing the ratio of acetoin/2,3-butanediol markedly to 3.01 from 1.02. These results demonstrated that the ability of *C. glabrata* to produce acetoin could be further enhanced through the regulation of intracellular NADH/NAD^+^.

## Discussion

As a pyruvate producer, the multi-auxotroph *C. glabrata* can be further engineered for acetoin production. However, compared with the larger accumulation of pyruvate, the acetoin titers produced are very low due to the inhibited pyruvate decarboxylation and intense spontaneous reaction of NOD, leaving a substantial range for improving acetoin production. Therefore, the GSMM *i* NX804 was employed as a starting point and additional reactions were included to account for the heterologous pathway. With the aid of *in silico* simulation, the heterologous pathway was evaluated as the more effective pathway for acetoin compared to the original pathway in *C. glabrata*. Thus, a novel pathway was designed and constructed in the cytosol, which was favorable for using pyruvate and transporting acetoin across the cytomembrane, making the production of acetoin 43-fold higher than that of the wild-type strain. This result demonstrated that GSMM *i* NX804 has predictive capability for metabolic engineering. Recently, the number of GSMMs available has sharply increased, and they are widely used to explore the space of microbial biochemical capabilities through promoting an understanding of metabolism on the global level [24-26]. For example, GSMM *i* NX804 was applied to identify the bottleneck for producing malate and provide a metabolic engineering strategy to significantly improve malate production in *C. glabrata*[27]. Thus, systems metabolic engineering opens a novel pathway for greatly improving the biochemical capabilities of microorganisms. However, due to the lack of model knowledge concerning microbial metabolism, regulatory mechanisms, and feedback inhibition, the application of GSMM has also been restricted, and requires further specific experimental investigation [17].

To construct a synthetic, composite pathway for acetoin production in *C. glabrata*, a three-step approach was employed in the present study. First, the candidate enzymes of ALS and ALDC were individually over-expressed and tested for enzymatic activity and the production of metabolites. Second, the best performing ALS and ALDC were co-expressed to construct a heterologous pathway in the cytosol, and evaluate their ability to produce acetoin. Finally, a push-and-pull strategy was adopted to achieve large flux amplification through the optimization of promoters. As a result, the concentrations of acetoin and 2,3-butanediol increased to 1.96 g/L and 1.32 g/L in the engineered *C. glabrata*, respectively. However, the production of ethanol also reached 3.68 g/L, which decreased the availability of pyruvate and offered another strategy for strain improvement.

To further improve acetoin production, the ethanol pathway was inhibited by disrupting the *ADH* gene. Interestingly, the production of acetoin was unchanged, but the level of 2,3-butanediol was increased by 53.8% along with the increase in the NADH/NAD^+^ ratio. As shown in Figure 1, the interconversion of acetoin and 2,3-butanediol is an NADH-dependent reaction, and high levels of NADH are favorable to form 2,3-butanediol. Thus regulating the level of intracellular NAD^+^ could be an alternative approach for further improving acetoin production. In previous studies, the water-forming NADH oxidase was employed to regulate the intracellular NAD^+^ level, achieving a highly efficient acetoin production [28,29]. Here, the precursor of NAD^+^, NA, was added to regulate the intracellular NADH/NAD^+^ ratio during acetoin fermentation, in which a lower level of intracellular NADH/NAD^+^ (0.56) was obtained, significantly inhibiting the reduction of acetoin and the biosynthesis of 2,3-butanediol. Consequently, the production of acetoin was further increased to 3.67 g/L, significantly increasing the ratio of acetoin/2,3-butanediol to 3.01 due to the insufficient reducing power [30,31]. However, the capacity to produce acetoin using *C. glabrata* was still far less than that of prokaryotes, such as *Serratia marcescens* H32 (75.2 g/L) [28], *Bacillus licheniformis* MEL09 (41.2 g/L) [32], and *Klebsiella pneumonia* (56.7 g/L) [3]. For example, *B. amyloliquefaciens*, a GRAS (generally regarded as safe) microorganism, exhibits a high yield (0.43 g/g glucose), productivity (1.42 g/(L · h)), and production (51.2 g/L) of acetoin during fed-batch fermentation [8]. Therefore, further enhancing the carbon flux of pyruvate into acetoin and blocking the reduction of acetoin, for example by disrupting butanediol dehydrogenase (BDH), could be an effective approach to improving acetoin production, and exploiting *C. glabrata* as a promising candidate for industrial acetoin production.

## Conclusions

In this study, we report the first production of acetoin by *C. glabrata* using the incorporation of systems metabolic engineering with cofactor engineering, significantly increasing the acetoin production to 3.67 g/L from trace amounts. However, a high concentration of pyruvate (15 g/L) also accumulated in the engineered strain, leaving substantial space for strain improvement. Actually, the carboligase reaction provides another pathway for acetoin production in yeast, in which pyruvate decarboxylase (PDC) catalyzes the irreversible non-oxidative decarboxylation of pyruvate and acetaldehyde to produce acetoin [33,34]. But for *C. glabrata*, pyruvate decarboxylation is strictly regulated by thiamine, and had become the rate-limiting step for producing acetoin. Therefore, increasing the activity of pyruvate decarboxylase and the availability of intracellular acetaldehyde may provide a novel opportunity to produce acetoin in a future study.

## Methods

### Strains and plasmids

*Escherichia coli* JM109 was purchased from Invitrogen and used for plasmid construction. The *C. glabrata* strains used were generated from *Candida glabrata* CCTCC M202019. More information about plasmids and strains is given in Table 4.

**Table 4:** Strains and plasmids used

**Strains/plasmids**	**Relevant characteristics**	**Reference**
**Plasmids**		
pY26	2 μm, Amp^R^, *URA3*, P_*GPD*_, P_*TEF*_	Lab. collection
pYX212	2 μm, Amp^R^, *URA3*, P_*TPI*_	Lab. collection
pY36	2 μm, Amp^R^, *URA3*, P_*TEF*_, P_*TEF*_	This study
pY16	2 μm, Amp^R^, *URA3*, P_*TPI*_, P_*TPI*_	This study
pY46	2 μm, Amp^R^, *URA3*, P_*GPD*_, P_*GPD*_	This study
pYX212-*BaALS*	Codon-optimized *ALS* from *B. amyloliquefaciens*	This study
pYX212-*BpALS*	Codon-optimized *ALS* from *Bacillus pumilus*	This study
pYX212-*BsALS*	Codon-optimized *ALS* from *B. subtilis*	This study
pYX212-*EcALS*	Codon-optimized *ALS* from *E. coli*	This study
pYX212-*BaALDC*	Codon-optimized *ALDC *from *B. amyloliquefaciens*	This study
pYX212-*BpALDC*	Codon-optimized* ALDC *from *B. pumilus*	This study
pYX212-*BsALDC*	Codon-optimized* ALDC *from *B. subtilis*	This study
pYX212-*EcALDC*	Codon-optimized* ALDC *from *E. coli*	This study
pY16-TPI-*BsALS*-TPI-*BaALDC*	Codon-optimized *ALS* and *ALDC* under TPI promoter	This study
pY26-TEF-*BsALS*-GPD-*BaALDC*	Codon-optimized* ALS* and* ALDC *under TEF and GPD promoter	This study
pY26-GPD-*BsALS*-TEF-*BaALDC*	Codon-optimized *ALS* and *ALDC *under GPD and TEF promoter	This study
pY36-TEF-*BsALS*-TEF-*BaALDC*	Codon-optimized* ALS* and* ALDC *under TEF promoter	This study
pY46-GPD-*BsALS*-GPD-*BaALDC*	Codon-optimized *ALS* and *ALDC *under GPD promoter	This study
**strains**		
*C. glabrata*CCTCC M202019	Multivitamin (thiamine, biotin, nicotinic acid and pyridoxine) auxotroph	[37]
*C. glabrata*Δ*ura*3Δ*Arg8*	The mutant derived from *C. glabrata* CCTCC M202019	[36]
MuA0	*C. glabrata *Δ*ura3*Δ*Arg8* (pYX212)	This study
MuA1	*C. glabrata *Δ*ura3*Δ*Arg8* (pYX212-*BaALS*)	This study
MuA2	*C. glabrata *Δ*ura3*Δ*Arg8* (pYX212-*BpALS*)	This study
MuA3	*C. glabrata *Δ*ura3*Δ*Arg8* (pYX212-*BsALS*)	This study
MuA4	*C. glabrata *Δ*ura3*Δ*Arg8 *(pYX212-*EcALS*)	This study
MuA5	*C. glabrata *Δ*ura3*Δ*Arg8* (pYX212-*BaALDC*)	This study
MuA6	*C. glabrata *Δ*ura3*Δ*Arg8* (pYX212-*BpALDC*)	This study
MuA7	*C. glabrata *Δ*ura3*Δ*Arg8* (pYX212-*BsALDC*)	This study
MuA8	*C. glabrata *Δ*ura3*Δ*Arg8* (pYX212-*EcALDC*)	This study
MuA9	*C. glabrata *Δ*ura3*Δ*Arg8* (pY16-TPI-*BsALS*-TPI-*BaALDC*)	This study
MuA10	*C. glabrata *Δ*ura3*Δ*Arg8* (pY46-GPD-*BsALS*-GPD-*BaALDC*)	This study
MuA11	*C. glabrata *Δ*ura3*Δ*Arg8* (pY36-TEF-*BsALS*-TEF-*BaALDC*)	This study
MuA12	*C. glabrata *Δ*ura3*Δ*Arg8* (pY26-GPD-*BsALS*-TEF-*BaALDC*)	This study
MuA13	*C. glabrata *Δ*ura3*Δ*Arg8* (pY26-TEF-*BsALS*-GPD-*BaALDC*)	This study
MuA14	*C. glabrata *Δ*ura3*Δ*Arg8*Δ*ilv5* (pY26-TEF-*BsALS*-GPD-*BaALDC*)	This study
MuA15	*C. glabrata *Δ*ura3*Δ*Arg8*Δ*ADH* (pY26-TEF-*BsALS*-GPD-*BaALDC*)	This study

### Plasmid construction and transformation

Standard cloning and bacterial transformations were performed according to Sambrook and Russell [35]. The following genes: *ALS* and *ALDC* from *B. subtilis*, *B. pumilus*, *B. amyloliquefaciens* and *E. coli*, were codon-optimized for expression in *C. glabrata* and synthesized by Sangon Biotechnology (Shanghai, China). These genes were gel extracted and inserted into the desired plasmid multi-cloning sites. In all cases, PCR was performed using TaKaRa *Pyrobest* DNA Polymerase (Takara Bio Inc, Shiga, Japan). All plasmids and genes were sequenced to ensure the correct identity of the insert prior to transformations. Yeast strains were transformed using the lithium acetate method [36].

### Construction of deletion strains

Inactivation of *ilv5* and *ADH* was achieved according to the method previously described [36]. The *ilv5*::*arg8* and *ADH*::*arg8* cassettes were constructed by fused PCR (Additional file 2), and transformed into the *C. glabrata* strain by electroporation. The transformants were identified as Arg^+^ prototrophic colonies on SM-U plates, and confirmed by colony PCR.

### Medium

During construction, strains were grown and maintained in Yeast peptone dextrose medium (YPD), minimal medium (MM), and supplement medium (SM) as follows. YPD: 10 g/L of yeast extract, 20 g/L of peptone, 20 g/L of glucose; MM: 20 g/L of glucose, 1.0 g/L of KH_2_PO_4_, 0.5 g/L of MgSO_4_, 10 g/L of (NH_4_)_2_SO_4_; and SM: MM with 80 mg/L of uracil or 40 mg/L of arginine (SM-U, for Δ *ura* 3 mutant; SM-A, for Δ *arg* 8 mutant); MM with 80 mg/L of uracil and 40 mg/L of arginine (SM-UA; for Δ *ura* 3 Δ *arg* 8 mutant); MM with 80 mg/L of branched-chain amino acids (valine, leucine, and isoleucine) and 30 mg/L of calcium pantothenate (SM-BP; for Δ *ilv* 5 mutant); and MM with 80 mg/L of uracil, 40 mg/L of arginine, 80 mg/L of branched-chain amino acids, and 30 mg/L of calcium pantothenate (SM-UABP; for Δ *ura* 3 Δ *arg* 8 Δ *ilv* 5 mutant).

During the fermentation, the medium for seed culture (medium A) consisting of 20 g/L glucose, 7 g/L urea, 5 g/L KH_2_PO_4_, 0.8 g/L MgSO_4_ · 7H_2_O, 3 g/L sodium acetate (15 g/L agar for solid medium); and the fermentation medium (medium B) containing 100 g/L of glucose, 3 g/L of urea, 7 g/L of KH_2_PO_4_, 0.8 g/L of MgSO_4_ · 7H_2_O, and 5 g/L of sodium acetate. The vitamin solution (8 mg/L of NA, 0.02 mg/L of thiamine, 0.04 mg/L of biotin, and 0.4 mg/L of pyridoxine-HCl) was added to all media (1% V/V). When necessary, the nutrients of uracil, arginine, branched-chain amino acids, and calcium pantothenate were added to overcome auxotrophy. Transformed *E. coli* JM109 cells were grown at 37 C in Luria-Bertani (LB) medium containing 100 μg/mL of ampicillin.

### Culture conditions

The seed culture was cultivated in a flask (25/250 mL medium A, 200 rpm, 30 C) for 24 h. Fermentation (50/500 mL medium B) was carried out in shake-flask culture (200 rpm, 30 C) using 40 g/L of CaCO_3_ as the buffering agent. Batch-fermentation was carried out in a 5-L jar fermenter (New Brunswick Scientific, Enfield, CT, USA) with 2.5 L of medium B (using 120 g/L glucose) under the conditions: the pH was controlled at 5.0 using 8 mol/L NaOH; the agitation speed and aeration rate were controlled at 400 rpm and 1.5 L/min; and performed at 30 C.

### Analytical methods

Cell growth was determined by measuring the optical density at 660 nm (OD_660_) using a UV–VIS spectrophotometer (Shimadzu UV mini 1240, Tokyo, Japan). The dry cell weight (DCW) was calculated using the DCW/OD_660_ ratio (DCW (g/L) = 0.23 OD_660_) according to a predetermined calibration line [37].

For extracellular metabolites analysis, the concentrations of glucose, ethanol, acetate, pyruvate, acetoin, and 2,3-butanediol were determined with a high performance liquid chromatography (HPLC) system (Dionex UltiMate 3000 Series, Thermo Scientific, USA) equipped with a Bio-Rad Aminex HPX-87H column (300 × 7.8 mm) and a refractive index detector (RID) [8]. Extraction and detection of intracellular NADH and NAD^+^ were carried out as previously described [38]. A 40-mL sample was collected and frozen in liquid nitrogen for 60 s, and then freeze-dried for 24 h and thawed in a solution containing 50 mM KOH, 30% ethanol, and 22 mM borate. After the pH was adjusted to 9.0-9.4 with 3 M HCl, extracted samples were centrifuged at 10,000 ×  *g* for 10 min, and then the supernatant was removed and its components were detected using HPLC. To analyze intracellular metabolites, samples were taken with a specialized rapid-sampling setup [39], and the intracellular metabolites were extracted with the method of freezing-thawing in methanol [40].

Determination of α-acetolactate and diacetyl was accomplished as described in previous reports [41] with minor modifications. Briefly, using 4 M H_2_SO_4_ to restrict the spontaneous decarboxylation of α-acetolactate to diacetyl before the analysis, and then the samples were examined using headspace gas chromatography with flame ionization detector (HS-GC-FID). During headspace at 70 C for 30 min in a low-pH environment, α-acetolactate was converted to acetoin, while diacetyl remained unchanged. Thus, α-acetolactate was quantified from the difference in the concentration of acetoin between the GC and HPLC results. HS-GC-FID analyses were performed using a gas chromatograph (GC-2010; Shimadzu Co., Kyoto, Japan), and analyte separation was accomplished on a capillary column (PEG-20 M, 30 m 0.32 mm I.D.). Helium was used as the carrier gas at a flow rate of 1.2 mL/min. The injection and detector temperatures were 200 C and 250 C, respectively, and the temperature program was as follows: 5 min at 40 C, subsequent increase to 180 C at the rate of 10 C per min, and 5 min at 180 C.

To measure ALDC and ALS activities, cells were harvested by centrifugation, and the extracts were prepared for assaying enzyme activity. ALDC activity was measured by the method of Loken and Stormer [42], and ALS activity was measured by the Atsumi method [41]. Protein concentration was determined by the Lowry method with bovine serum albumin as the standard [43].

### *In silico* simulation

The impact of a heterologous pathway on cell growth and acetoin production was simulated *in silico* by flux balance analysis (FBA) using the GSMM of *C. glabrata i* NX804, comprised of 804 genes, 1287 reactions, and 1025 metabolites (Additional file 1b) [15], in which the reactions representing the heterologous enzymatic activities of ALS and ALDC were added to the model. First, maximum cell growth was set as the initial objective in the modified model. Subsequently, the theoretical maximum value of acetoin production was obtained using acetoin as the objective when the cell growth was decreased to 90% μ_max_. FBA simulations were performed using COBRA Toolbox-2.0 through the MATLAB interface, with GLPK as the linear programming solver [44].

## Abbreviations

ALS: Acetolactate synthase; ALDC: Acetolactate decarboxylase; GSMM: Genome-scale metabolic model; NOD: Non-enzymatic oxidative decarboxylation; BDH: Butanediol dehydrogenase; PDC: Pyruvate decarboxylase; NA: Nicotinic acid; BCAAs: Branched chain amino acids; 3-PG: 3-phosphate-glyceraldehyde.

## Competing interests

The authors declared that they have no competing interests.

## Authors’ contributions

SBL and LML conceived the study. SBL made contribution to the design of the experiments, the acquisition of data, the analysis and interpretation of data and contributed to the manuscript writing. XG helped to construct the engineered strains. NX provided the *in silico* simulation data. LML and JC conceived and organized the study and helped to draft the manuscript, and has revised the manuscript. All the authors read and approved the final manuscript.

## Supplementary Material

Additional file 1a **Additional descriptions for Figure **2**.** A list of the corresponding enzymes, reaction equations and subsystems for target reaction listed in Table S1. b: Additional descriptions for Figure 2. A list of additional description of the model *i* NX804 for *in silico* simulation. Click here for file

Additional file 2 The construction of deletion cassettes for Figure S1. Click here for file
