# Supplementary material for: Enhancement of acetoin production in Candida glabrata by in silico-aided metabolic engineering
Source: Microb Cell Fact. 2014 Apr 13;13:55. doi: 10.1186/1475-2859-13-55 (PMC4021295; doi:10.1186/1475-2859-13-55)
Supplement: Additional file 1a — Additional descriptions for Figure 2. A list of the corresponding enzymes, reaction equations and subsystems for target reaction listed in Table S1. b: Additional descriptions for Figure 2. A list of additional description of the model i NX804 for in silico simulation. [file 1475-2859-13-55-S1.zip › add1/6143602251169071_add1.docx]

Table S1 Reactions added to the*i*NX804 model to account for the heterologous acetoin pathway

| Reaction name | Reaction formula* |
| --- | --- |
| ‘ALS’ | 2HTPP[c] + PYR[c] + H[c] ⬄ ACLAC[c] + TPP[c] |
| ‘ALDC’ | ACLAC[c] ⬄ACT[c] + CO_2_[c] |
| ‘ACT e’ | ACT[c] ⬄ ACT[e] |
| ‘EX-ACT’ | ACT[e] ⬄ |

*****The following abbreviations were used: ALS, α-acetolactate synthetase; ALDC, α-acetolactate decarboxylase; ACT, acetoin; PYR, pyruvate; ACLAC, α-acetolactate; TPP, diphosphothiamine. [c] and [e] denote the cytoplasm and extracellular compartments, respectively.
