# Supplementary material for: Enhancement of acetoin production in Candida glabrata by in silico-aided metabolic engineering
Source: Microb Cell Fact. 2014 Apr 13;13:55. doi: 10.1186/1475-2859-13-55 (PMC4021295; doi:10.1186/1475-2859-13-55)
Supplement: Additional file 2 — The construction of deletion cassettes for Figure S1. [file 1475-2859-13-55-S2.docx]

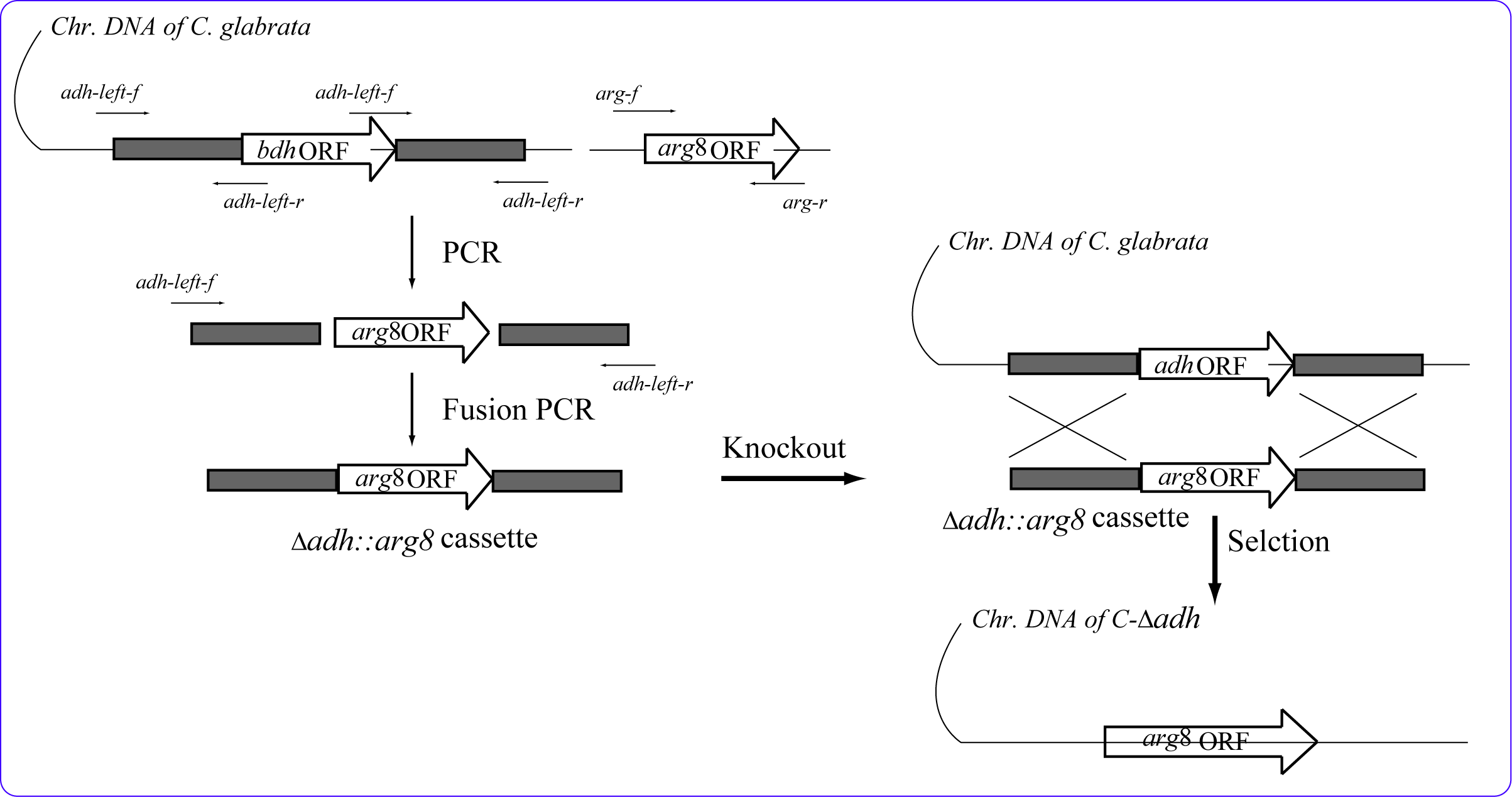


Fig. S1 The deletion of gene *adh* using the construction of fusion frames. Similarly, the deletion of gene *ilv5* was also carried out in strain MuA13.
